# Society Proceedings

**Published:** 1903-04

**Authors:** 


### SOCIETY PROCEEDINGS.

### WISCONSIN SOCIETY OF VETERINARY GRADUATES.

THE annual meeting of the Society was held in the club rooms of the Avenue Hotel, Madison, at 3 P. M., February 4, 1903.

The meeting was called to order by the President, J. F. Roub, and the following members answered to roll-call: S. Beattie, J. W. Beckwith, C. M. Crane, C. E. Evans, H. F. Eckert, G. H. Fay, L. M. Jargo, G. E. Leech, W. J. Malone, E. H. Newton, J. F. Roub, E. D. Roberts, D. Roberts, T. A. Schneekloth, Charles Schmitt, S. S. Snyder, and one visitor, Dr. A. J. Alexander.

The minutes of the last meeting were read and adopted.

The Secretary's report was read and accepted.

The Treasurer's report was read and accepted.

On motion, an auditing committee was appointed by the Chair to examine the books of the Secretary and the Treasurer.

The President appointed Drs. Schmitt, Evans, and Beckwith, who examined the books and signed them as correct.

Dr. G. H. Fay, an early member of this Society, who had discontinued practice for a time, applied for admission as a new member.

It was moved and seconded that Dr. Fay be accepted as a new member by paying a membership fee of $3.00 and one annual due of $2.00 for the year 1903. Carried.

Dr. Fay responded by thanking the Society for the action it had taken, and promised to become an active member.

The subject of holding our next semi-annual meeting in Milwaukee was discussed in a lively manner by Drs. Schmitt, Evans, Roub, and Beckwith, which resulted in a motion, that was seconded, that we hold our next meeting in Racine. Carried.

Dr. Alexander addressed the meeting with a nice little talk on horse-feed and horse-feeding, which he sweetened at the beginning with molasses, but as the doctor proceeded to illustrate the disadvantage of using molasses as food for horses, and its inferior value as compared with good oats, that sweetness turned to bitterness in a decision against the molasses.

On motion, a vote of thanks was extended to Dr. Alexander.

On motion, the Society adjourned to meet at 7.30 P.M.

### EVENING SESSION.

The Secretary read a bill, offered by State Veterinarian Roberts, relative to prohibiting the importation of Western-branded horses into Wisconsin without a veterinary inspection, as a great deal of glanders and other contagious diseases is brought East by this class of horses, and a change was proposed that, in place of Western-branded horses, it should read Western horses, branded and otherwise.

The President, in view of the fact that several members had violated the code of ethics by placing on the market proprietary remedies, asked the

Secretary to read the code, which was read and discussed by all the members present.

Motion was made and seconded that the Chair appoint a committee of three members to revise the code of ethics and make it more lenient. Carried. The Chair appointed Drs. R. S. Heer, G. H. Fay, and S. S. Snyder.

It was moved and seconded that the committee report at our next meeting. Carried.

The Secretary read a bill, framed by the Committee on Legislation, for the purpose of establishing a Veterinary Examining Board; also a letter from Superintendent Whitehead advising the substitution of medical doctors on that board in place of veterinarians. On motion, the Secretary was instructed to answer Mr. Whitehead's letter, declining to change the original bill.

Dr. R. S. Heer read an excellent paper on "External Ulcerative Anovulvitis in Cattle," which was discussed by Drs. D. Roberts, Alexander, Leech, and Schmitt. Dr. Beckwith reported a similar outbreak in his own herd.

Dr. Snyder reported a peculiar case of influenza in a horse in which the animal had intermittent attacks of digestive trouble for several weeks, which was discussed by Drs. D. Roberts, Alexander, and Leech.

On motion, the Society proceeded to the election of officers, which resulted as follows: President, Dr. S. S. Snyder, Cedarburg; Vice-President, Dr. R. S. Heer, Platteville; Secretary, S. Beattie, Madison; Treasurer, Dr. Charles Schmitt, Dodgeville; Censors, Drs. J. W. Beckwith. G. H. Fay, and H. F. Eckert.

Dr. E. D. Roberts reported several outbreaks of rabies throughout the State, which was discussed by several members.

Dr. Leech, who is an honorary member, moved that the Society accept his usual annual dues. The m otion was seconded and carried.

Motion was made and seconded that the Society uphold the just acts of the State veterinarian. Carried.

On motion, the Society adjourned to meet at Racine, subject to the call of the President and Secretary.

S. Beattie,
Secretary.

---

### PENNSYLVANIA STATE VETERINARY MEDICAL ASSOCIATION.

The annual meeting was held at the Odd Fellows' Temple, Broad and Cherry streets, Philadelphia, March 3 and 4, 1903, President W. L. Rhoads in the chair, the following members and visitors being present:

*Members:* N. H. Allis, Wyalusing; F. S. Allen, Philadelphia; W. G. Benner, Doylestown; Henry Bowers, Collegeville; John Bradley, Mercersburg; J. F. Butterfield, South Montrose; T. S. Carlisle, Chestnut Hill; H. B. Cox, Philadelphia; M. E. Conard, West Grove; John M. Courtright, Clark's Green; M. J. Collins, Myerstown; D. A. Diemer, Spring City; H. P. Eves, Wilmington, Del.; H. B. Felton, Olney; Frank W. Fernsler, Lebanon; J. C. Foelker, Allentown; W. H. Fry, Pine Grove Mills; G. M.

Fuller, Philadelphia; E. A. Gruver, Richlandtown; S. J. J. Harger, Philadelphia; F. F. Hoffman, Brookville; W. Horace Hoskins, Philadelphia; J. S. Houldsworth, Manayunk; H. P. Keely, Schwenksville; Morris W. Keck, Slatington; W. S. Kooker, Philadelphia; W. H. Knight, Kennett Square; Charles Lintz, Chester; E. S. Moyer, Blooming Glen; C. J. Marshall, Philadelphia; J. C. McNeil, Pittsburg; H. D. Martien, Philadelphia; L. E. Meade, Tunkhannock; W. H. Mattson, Camp Ground; M. Moriarity, Gettysburg; Otto G. Noack, Reading; George Magee, Uniontown; O. M. Norton, Coatesville; Leonard Pearson, Philadelphia; E. M. Ranck, Glenolden; James B. Rayner, West Chester; J. B. Raynor, Milestown; W. L. Rhoads, Lansdowne; W. H. Ridge, Trevose; Frank Standen, Philadelphia; A. F. Schreiber, Philadelphia; B. F. Senseman, Philadelphia; E. K. Shaub, Lancaster; E. E. Tower, Montrose; J. H. Timberman, Wilkesbarre; E. E. Terry, Holmesburg; H. W. Turner, Lahaska; Benjamin Underhill, Media; V. B. Weaver, Center Valley; A. W. Weir, Greenville; B. T. Woodward, Oxford.

*Visitors:* H. S. Stoker, H. M. Cullen, E. S. Dembler, C. M. Hench, T. J. Mahaffy, C. H. Woodruff, S. T. Young, N. B. Fleming, S. Reed, H. E. States, R. F. Burton, G. W. Teufel, J. B. Tiffany, J. N. Becker, G. H. Hart, W. D. Fulton, J. H. Morse, D. E. Hickman, H. B. Hood, Dr. Harshberger, Frederick Stehle, A. H. Shields, H. K. Copithorn, Henry Jarrett, F. H. Collins, A. H. Metzger, Mark White, Jr., S. W. Nisley.

President Rhoads delivered his annual address in his usual interesting manner.

The following officers were regularly nominated and elected for the ensuing year:

*President:* Dr. E. M. Ranck.

*Vice Presidents:* Drs. F. F. Hoffman, J. C. McNeil, and J, H. Timberman.

*Treasurer:* Dr. Francis Bridge.

*Recording Secretary:* Dr. H. B. Felton.

*Corresponding Secretary:* Dr. C. J. Marshall.

*Trustees:* Drs. Leonard Pearson, James B. Rayner, W. Horace Hoskins, George B. Jobson, and Jacob Helmer.

Meeting adjourned for lunch till 2 o'clock.

The following names were proposed for membership: Drs. L. E. Meade, F. W. Fernsler, B. T. Woodward, F. H. Standen, H. D. Martien, S. H. Gilliland, O. M. Norton, John Henry Farley, and James H. St. Clair. These applications were referred to the Board of Trustees. The board reported favorably on all except Drs. Farley and St. Clair. These names were to be returned to the applicants for the proper vouchers. The other applicants were regularly elected to membership.

In the absence of some of the members of the Board of Trustees the President appointed the following as temporary members of this board: Drs. H. B. Felton, B. M. Underhill, W. H. Ridge, W. S. Kooker, and F. S. Allen.

Dr. E. M. Ranck read his report as Corresponding Secretary. It was regularly accepted and filed. Dr. Ranck also read a letter from Dr. J. C. Michener, of Colmar, submitting his resignation. The letter was referred to the Board of Trustees. They recommended that his resignation be accepted and that he be elected to honorary membership. This was unanimously adopted.

The Treasurer's report was read by the President. It was regularly accepted and filed.

In the absence of Dr. George B. Jobson, Secretary of the Committee on Legislation, this report was postponed till later in the meeting.

Dr. Hoskins made a verbal report for the Committee on Army Legislation. He said, in part, that very little had been done in the past three years on this subject, yet the general status of the veterinarians in the army had been markedly improved by the work that has been done. He thinks that official recognition with rank is a possibility of the near future.

Dr. Noack made a few remarks on Dr. Hoskins' report. He thinks that one important move in getting the proper recognition would be for the veterinary colleges to raise their requirements for entrance.

The Committee on Sanitary Science and Police made no report.

A carefully prepared report was made by Dr. Helmer for the Committee on Milk Inspection, which was unanimously accepted and filed with the Secretary. Dr. M. E. Conard, as a member of this committee, made an interesting verbal report.

The Committee on Intelligence and Education, Dr. H. B. Felton, chairman of this committee, gave an interesting and carefully prepared report, as follows:

*Report of Committee on Intelligence and Education.*

The past year has been one of phenomenal prosperity in the business world. Sharing in this prosperity the veterinarian has had no cause to complain of dull practice or lack of adequate compensation. Despite the threat of the automobile, the horse is still with us in large and imposing numbers and with an average monetary value so great that his owner dare not discard the services of the veterinarian. The horse is still the faithful and efficient servant of the Government in carrying the mails and in the cavalry and artillery service. In Berlin, Germany, recently they became very aggressive, and displaced the horse by the electric automobile in the mail service. After a few weeks' trial, however, it was found that the electric automobile, in all cases, got out of order, and the postal officials, becoming tired of sending horse-wagons after the automobiles, finally discarded them altogether.

In reference to the use of the horse in the army, for the first time in the history of our country the proper treatment of the horse is made the subject of comment and suggestion in a President's message. Mr. Roosevelt asks Congress to make an appropriation to enable the Secretary of War to keep and care for cavalry and artillery horses that have worn out in long service. The friends of our friend, the horse, are everywhere commenting favorably on the suggestion and on the President's thoughtful kindness.

Agriculture is so intimately connected with our profession that this report would not be complete without some reference being made to this subject. We hear much in these days of gigantic corporations with vast aggregations of capital, and when the United States Steel Company was organized recently our eyes were dazzled and our brains made to swim when it was capitalized at one billion dollars. In the meantime the farmer, the man of the hoe and plow, the plain man, the horny-handed son of toil, who earns his bread by the sweat of his brow, was making no boasts or no display, but when the aggregate amount of his invested capital was computed it was found to reach the stupendous sum of twenty billions of dollars. The farmer is still king, and he can walk with majestic strides in the company of the gigantic trusts, towering far above them all and making them look like thirty cents! There are about 5,000,000 farms under cultivation in this country, comprising an area of 415,000 square miles. A much larger area is susceptible of culture, and awaits the advent of the settler to make the wilderness rejoice and blossom as the rose. Forty millions of people, more than one-half the population of the country, reside on farms. The products of American agriculture aggregate annually over $5,000,000,000. Animals annually slaughtered for food possess a value of nearly $1,000,000,-000. Dairy products are worth over $500,000,000; poultry and eggs more than $300,000,000. Dairy products have found new markets during the past year in China, Japan, Cuba, and Porto Rico. Recognizing the great importance of agriculture to the welfare of our country, the Government is spending not less than $5,000,000 yearly, and employing an army of experts who are engaged in experimental work and who are searching in all quarters of the globe for new grasses, grains, and fruits. Grasses and forage crops are diligently studied with reference to their adaptation in different sections of the Republic. Not one or two, but many blades of grass will be made to flourish where none is growing now. A special study is being made of soils and their relation to crops. Experiments in feeding are being carried out with a view to economy and securing better results for the labor and skill applied. Bulletins are published from time to time giving the results of experimental work, and these are freely accessible to the farmer. With a generous paternalism the Government is continuing the Congressional distribution of seeds, and particularly of foreign seeds, over a constantly-enlarging area, and with gratifying results. The farmer of to-day is more of a scientific farmer than ever before, and has come to realize that farming can be made to pay when conducted on strictly business lines. Electricity has become his servant, and we see stretching over the country a network of telephone wires, connecting the farmer with his neighbor and with the business world, thus removing his isolation and enabling him to keep his finger on the pulse of the market. It has become an axiom in certaim localities, "If you want to know the news, ask the farmer."

Perhaps more improvements have been made along all lines than along the line of good roads. As Secretary Wilson has observed, "We still need to mend our ways." Why should millions of dollars be spent every year by the great railroad systems for betterments, and millions of dollars be appropriated by the Government for river and harbor improvements, and our highways be neglected, when they are such an important part of the great system of transportation in our country? It is a question of educating the people up to a point where they will realize the great importance of this subject, and we are glad to know that the Federal Government and a number of States are carrying on this campaign of education. The Government has built sample roads in Maryland, West Virginia, Ohio, and North Carolina. In co-operation with the Southern Railway Company it has done educational work in Virginia, North Carolina, Tennessee, Alabama, and Georgia. The railroad company assisted in this because of its conviction that good roads develop the country, and it is therefore helpful to the business of a railroad to have good country roads leading to its stations. We could wish that this idea might find lodgement in the heads of the managers of the Pennsylvania and Reading railroads, for, by their combined efforts, they might do wonders to help along the good cause in Pennsylvania. We have a good roads law on the statute books, which was passed several years ago, but its efficiency has never been tested, because its going into operation is made contingent on the Legislature appropriating $1,000,000 for it, and that provision has acted as a bar to its enforcement. Much is hoped for from the Sproul bill, which will soon be acted upon. There seems to be a disposition, however, in this State to look at the subject of road improvement from entirely too narrow a standpoint, one man being fearful that perhaps his neighbor at a distance will receive more benefit from the tax than he will. There is a need of a broader outlook and a recognition of the fact that good roads, no matter where situated, are a benefit to the whole State. The farmer is finding it to be increasingly profitable to breed horses that conform closely to a certain type, and valuable information on this subject to the farmer, as well as to the veterinarian, will be found in Bulletin No. 37, issued by the Bureau of Animal Industry, entitled *Market Classes of Horses*, by George M. Rommel, B.S.A.

No widespread epidemics among animals has visited our country during the past year. Our island possessions have not been so fortunate, however, as an epidemic of rinderpest in the Philippines swept away 90 per cent. of the buffaloes, which are there used as beasts of burden. Foot-and-mouth disease broke out in Massachusetts, but, owing to the rigid and effective quarantine, did not spread beyond the borders of that State.

The efficiency of the process of Pasteurization of milk in preventing disease among infants and small children has been remarked by the New York city health authorities, who state that during the past year there has been a decided decrease in infant mortality in districts accessible to the depots where Mr. Nathan Straus' philanthropy makes it possible for parents in the tenement houses to get pasteurized milk gratuitously or at a nominal cost.

The fight against tuberculosis is being carried on with increasing vigor and greater effectiveness. No less than eleven States have actively taken up the subject of the prevention of tuberculosis. The Pennsylvania Society for the Prevention of Tuberculosis has prepared a bill asking for an appropriation from the State of $500,000 for the prevention of tuberculosis and the care of incipient cases of consumption in this State. It is proposed to establish sanatoria for the care of poor consumptives in different parts of the State similar to the one at White Haven, where such excellent results have been obtained. As Dr. Anders says, the Government should recognize and seize the opportunity to do this work. He further observes, "the State already provides for the insane, for the feeble-minded, for the defectives, for the deaf and dumb;" yet these do not constitute one-third the number of consumptives. It may almost be said that for selfish reasons alone it is the State's duty to care for poor consumptives. If cholera, smallpox, scarlet fever, or any of the dreaded contagious diseases caused one-fourth the deaths claimed by consumption, remedial measures would be at once taken by the State; and yet consumption kills more than all these contagious diseases combined. England and Germany are becoming more fully awakened to the possibilities of coping with this disease and the economy exercised thereby. Germany has sixty-four sanatoria, and it is said that in fifteen of them 60 per cent. of the patients were discharged as cured. One valuable feature in connection with the sanatoria in Germany is the establishment of convalescent homes, where they are allowed to stay two or three months, thus obtaining a more permanent cure. One of the grandest gifts of recent times is the noble benefaction of Mr. Henry Phipps of $1,000,000 for establishing a hospital for consumptives in Philadelphia. This has long been a crying need in our great city, as none of the hospitals want this class of patients, and, after making the rounds of the different hospitals, in each of which they are only allowed to remain a short time, they virtually become outcasts, and are left to die in homes of poverty, lacking the care and attention that would soothe their last hours and render their lot less pathetic.

The State Live-stock Sanitary Board has done excellent work during the last year, but has been much hampered by lack of funds to prosecute its work. It is to be hoped that it will receive a liberal appropriation for the ensuing year. The exceedingly valuable experimental work of Drs. Leonard Pearson and S. H. Gilliland on the "Immunization of Cattle against Tuberculosis" places our State in the very front rank of those engaged in original work on this subject. Dr. Pearson hopes the State Board will receive an appropriation large enough to enable it to establish a tubercular herd, where experiments can be carried out under natural farm conditions. Dr. E. M. Ranck is at the present time making a study of the organisms that cause distemper, or rhinoadenitis, shipping-fever, and influenza in horses. If he can discover an effective antitoxin he will place the raiser, shipper, and purchaser of horses under lasting obligations to him, as well as adding lustre to our profession.

The Veterinary Department of the University of Pennsylvania has been placed under considerable disadvantage while residing in temporary quarters. We are informed that the architect's plans are now practically finished, and building will commence just as soon as the funds in Mr. Harrison's hands will justify it. A bill has been introduced in Harrisburg to appropriate $200,000 to the University, $100,000 of which is for the Veterinary Department. The outlook for research and original investigation, especially along medical lines, was never so bright as at present with the Carnegie University at Washington and the Rockefeller Institute in New York, magnificently endowed by the millions of their generous donors to stimulate the efforts of the brightest minds our country can produce. By the recent establishment of the several universities—national in their scope—in our country's capital Washington will become the greatest educational centre in the United States, if not in all the world. The standard of education and the requirements of those seeking admission to the professional schools are being gradually raised by our colleges and universities. President Eliot has well said: "Since wise and efficient conduct of American affairs, commercial, industrial, and public, depends more and more upon the learned and scientific professions, the universities owe it to the country to provide the best possible preparation for all these professions." Chancellor Smith, of Randolph-Macon College, has made some very interesting and valuable investigations on the power of education to raise a man above the common lot. He finds that the uneducated person has only one chance in 150,000 of attaining distinction. A common-school education will increase his chances nearly four times. A high-school education will increase his chances twenty-three times over the common-school boy, or nearly ninety times over the uneducated one. The college education increases his chances over the high-school boy about nine times, over the common-school boy about two hundred and twenty times, and over the uneducated boy more than eight hundred times.

We cannot close without referring to the sense of gratification we are sure every member of our profession in this State feels at the reappointment of Dr. Pearson as State Veterinarian. Dr. Pearson has filled this position with such conspicuous ability that his name and his fame have become international. We also feel constrained to refer to the noble and heroic efforts of Dr. Hoskins to maintain the publication of the JOURNAL in the face of so many adverse circumstances. The JOURNAL is steadily maintained as a strictly first-class publication in every respect, and is excelled by none. It deserves the hearty support of every member of our profession.

H. B. FELTON, V.M.D.,
Chairman.

Dr. Noack, as a member of this committee, made a few appropriate remarks.

The Publication Committee made no report.

---

### CONVENTION ECHOES.

Dr. Edgar M. Powell, of Bryn Mawr, Pa., was confined to his bed during the convention sessions.

Dr. C. R. Good, of Lock Haven, Pa., reported his county free from any contagious and infectious diseases, and also great interest in good roads.

The tender of the resignation of Dr. J. Curtis Michener was regretfully received, and his election to honorary membership was a well-merited recognition of valuable work done.

The election to active membership of Drs. S. H. Gilliland, B. S. Woodward, H. D. Martein, H. P. Fernsler, Frank Standen and L. E. Mead will add new strength to the State organization.

The attendance was well distributed from the many sections of the State, about one-half the counties having representation at the convention.

Delaware was represented by the presence of Dr. H. P. Eves, of Wilmington.

Dr. W. H. Fry, of Pine Grove Mills, Pa., was Chairman of the General Committee conducting the Centre County Farmers' Institute in January.

Dr. W. P. Bagnall, of Warren, Pa., has been appointed Inspector of Dairies for his city.

